# Comparison of Printable Biomaterials for Use in Neural Tissue Engineering: An In Vitro Characterization and In Vivo Biocompatibility Assessment

**DOI:** 10.3390/polym16101426

**Published:** 2024-05-17

**Authors:** Miguel Etayo-Escanilla, Noelia Campillo, Paula Ávila-Fernández, José Manuel Baena, Jesús Chato-Astrain, Fernando Campos, David Sánchez-Porras, Óscar Darío García-García, Víctor Carriel

**Affiliations:** 1 Department of Histology, Tissue Engineering Group, University of Granada, 18016 Granada, Spain; metayo@ugr.es (M.E.-E.); pavila@ugr.es (P.Á.-F.); fcampos@ugr.es (F.C.); vcarriel@ugr.es (V.C.); 2 Instituto de Investigación Biosanitaria ibs.GRANADA, 18012 Granada, Spain; 3 Doctoral Program in Biomedicine, University of Granada, 18012 Granada, Spain; 4 REGEMAT 3D, Avenida Del Conocimiento 41, A-111, 18016 Granada, Spainjm.baena@regemat3d.com (J.M.B.); 5 BRECA Health Care S.L., Avenida Del Conocimiento 41, 18016 Granada, Spain

**Keywords:** neural tissue engineering, biomaterials, 3D printing, scaffolds, biocompatibility, biomechanic

## Abstract

Nervous system traumatic injuries are prevalent in our society, with a significant socioeconomic impact. Due to the highly complex structure of the neural tissue, the treatment of these injuries is still a challenge. Recently, 3D printing has emerged as a promising alternative for producing biomimetic scaffolds, which can lead to the restoration of neural tissue function. The objective of this work was to compare different biomaterials for generating 3D-printed scaffolds for use in neural tissue engineering. For this purpose, four thermoplastic biomaterials, ((polylactic acid) (PLA), polycaprolactone (PCL), Filaflex (FF) (assessed here for the first time for biomedical purposes), and Flexdym (FD)) and gelatin methacrylate (GelMA) hydrogel were subjected to printability and mechanical tests, in vitro cell–biomaterial interaction analyses, and in vivo biocompatibility assessment. The thermoplastics showed superior printing results in terms of resolution and shape fidelity, whereas FD and GelMA revealed great viscoelastic properties. GelMA demonstrated a greater cell viability index after 7 days of in vitro cell culture. Moreover, all groups displayed connective tissue encapsulation, with some inflammatory cells around the scaffolds after 10 days of in vivo implantation. Future studies will determine the usefulness and in vivo therapeutic efficacy of novel neural substitutes based on the use of these 3D-printed scaffolds.

## 1. Introduction

According to the findings from the Global Burden of Diseases, Injuries, and Risk Factors Study, traumatic injuries to the nervous system are the main cause of morbidity, disability, and mortality among young adults in industrialized nations [1]. On one hand, the estimated prevalence of spinal cord injury (SCI) ranges between 236 and 1009 per million of the population [2]. On the other hand, the incidence of peripheral nerve injury (PNI) has been estimated to be from 130 to 230 per million of the population per year [3]. Nervous system injuries are normally associated with the progressive damage of neural cells, affecting the patient’s quality of life and causing socioeconomic impacts on healthcare systems worldwide [4].

In the peripheral nervous system, small nerve transections are commonly treated through direct reconnection (neurorrhaphy), while injuries with a larger nerve gap are typically addressed using the nerve autografts technique, which is still considered the “gold standard” treatment in the field. However, morbidity at the donor site, a lack of graft material, and extended surgical times are well-known limiting reasons for the use of autografts [5]. Attempts to replace autologous nerve grafts with allografts and xenografts were made, but they were discontinued due to concerns about immune rejection and the potential for disease transmission [6]. Repairing SCI is really challenging, due to the limited regenerative capacity of the central nervous system [7]. One treatment for SCI involves administering high doses of methylprednisolone to reduce secondary damage; however, this therapy causes many critical side effects and does not yield substantial neurological improvements. Other therapeutic options are surgical interventions to anatomically stabilize and decompress the spinal cord and rehabilitative care, which have modest outcomes [8]. For these reasons, researchers and clinicians have been collaborating over the last decades to explore innovative strategies for treating nervous system injuries, and the fields of tissue engineering (TE) and regenerative medicine have emerged, with promising results.

TE is a multidisciplinary field that applies principles from engineering and life sciences to develop, on one hand, biological substitutes that could restore or maintain tissues and organs functions [9] and, on the other hand, human-based models for both fundamental and preclinical research [10]. Over the past few decades, TE has achieved impressive advancements, and now it plays a crucial role in developing therapies for patients afflicted with severe chronic diseases affecting vital organs such as the kidneys, heart, and liver [11]. Moreover, TE is also contributing to progress in treating various other conditions, including skin burns [12], corneal replacement [13], and nervous system repair [14], among others.

Neural tissue engineering (NTE) has a specific focus on creating advanced and biomimetic scaffolds that offer a supportive three-dimensional (3D) structure for cell adhesion, growth, differentiation, and the transport of biological substances [15]. An ideal scaffold for NTE should be biocompatible to provide cell adhesion, proliferation, and differentiation, and it should present appropriate mechanical properties to prevent increased stress in the lesion region or collapse throughout regular motion [16].

Numerous natural, synthetic, and hybrid biomaterials have been investigated for the development of suitable scaffolds aimed at restoring neurological functions [8]. The main advantage of using naturally derived biomaterials is that they offer the relevant biomimetic microenvironments to cells, since they retain the essential components and cell signaling cues found in the cellular niche. However, they provide limited control over the mechanical and structural properties of the scaffold. Alternatively, synthetic polymers offer great control over the scaffold architecture and tunable mechanical properties, however, they tend to be less biocompatible and often require additional engineering, such as the incorporation of binding sites [17]. Frequently, a combination of natural and synthetic polymers is investigated, where natural polymers provide a biomimetic environment for cells and synthetic polymers suitable structural and mechanical support [18].

Different methods have been employed for the production of 3D scaffolds, such as gas foaming, melt molding, electrospinning, and phase separation. However, these methods do not provide the flexibility to adjust the scaffold shape, inner channel configuration, or pore size [19]. In contrast, 3D printing technology has evolved over the last century as a promising alternative to create, through an additive manufacturing manner, complex 3D constructs by precisely controlling its architecture (external shape, internal pore geometry, and interconnectivity), with high reproducibility and repeatability. Three-dimensional printing technology has developed into a variety of printing methods, such as inkjet, stereolithography, and the widely used extrusion [15]. Extrusion printing involves layer-by-layer deposition through a micronozzle controlled with a computer. It is divided into different fusion-based processes (e.g., fused deposition modeling (FDM)) and dissolution-based processes (e.g., 3D plotting). While this technique offers relatively lower resolution, it is considered promising due to its cost-effectiveness, speed, and ability to generate organized constructs of clinically relevant size within a reasonable timeframe [16,20].

Taking all of these factors into consideration, the aim of this study was to evaluate and select the most suitable biomaterials for applications in NTE using extrusion-based 3D printing. The chosen biomaterials included different thermoplastics, from the most widely studied ones, such as polylactic acid) (PLA) and polycaprolactone (PCL), to the less widely explored conductive Filaflex (FF) and Flexdym (FD), and finally a gelatin methacrylate hydrogel (GelMA). PLA is an FDA-approved synthetic biodegradable polymer that can be produced from the renewable feedstocks of sugar cane and corn [21]. This material exhibits desirable mechanical characteristics, such as excellent thermal stability and degradability, which make it suitable for TE applications [22]. PCL is also an FDA-approved synthetic biodegradable polymer, which is currently derived from fossil fuels on an industrial scale [23]. It is characterized by its mechanical elasticity, long-term degradation, low melting temperature, and its stable nature within a living body; moreover, it has been widely used in clinical applications [22]. Both PLA and PCL have been used, experimentally, in NTE, obtaining promising results, especially in the generation of nerve guide conduits [24,25,26,27,28]. GelMA is a photocrosslinkable gelatin-based hydrogel that has attracted considerable attention in the field of tissue engineering [29,30,31], as it offers the synergistic effects of biofunctionality and mechanical tunability. The bio-functionality of GelMA is possible due to the presence of the bioactive motifs of gelatin, derived from collagen, while the mechanical properties could be easily tuned by varying the polymer compositions, the polymer concentration, and the crosslinking intensity [32]. FF, on the other hand, is a newly developed thermoplastic polyurethane with high flexibility (92A shore hardness) and electroconductive properties, according to its manufacturer [33]. Although it has been employed in the field of electronics, its potential for use in NTE applications has yet to be fully explored. FD is also an innovative thermoplastic elastomer that has been investigated for microfluidics applications. [34]. It is flexible, stretchable, and biocompatible, which makes it a promising biomaterial for NTE [35]. Thus, this study aimed to evaluate and compare the aforementioned biomaterials under the same experimental conditions. Here, they were subjected to printability tests, and the generated 3D-printed scaffolds to mechanical characterization, an in vitro cell–biomaterial interaction analysis, and an in vivo biocompatibility assessment.

## 2. Materials and Methods

### 2.1. Preparation of Biomaterials for 3D Printing

The following thermoplastics were employed in the fabrication of the 3D-printed scaffolds: polylactic acid filament (96% L-isomer) (PLA Ivory White filament, Smart Materials 3D, Jaén, Spain), polycaprolactone homopolymer filament with a molecular weight of 50.000 g/mol (Facilan^TM^ PCL 100 filament, 3D4MAKERS, Haarlem, The Netherlands), conductive Filaflex filament (10% LampBlack additive) (RECREUS, Alicante, Spain), and Flexdym pellets (combination of styrene–(ethylene/butylene)–styrene (SEBS)) (Eden Microfluidics, Paris, France), all of which were in a ready-to-use format.

Additionally, a hydrogel composed of lyophilized gelatin modified with methacryloyl groups (75–85% of methacrilation degree) (GelMA Claro^®^ BG800, PB Leiner. Part of Tessenderlo Group, Brussels, Belgium) and the crosslinker lithium phenyl-2,4,6-trimethylbenzoylphosphinate (LAP, TCI Chemicals, Tokyo, Japan) were employed. For the preparation of 10% GelMA/0.5 LAP hydrogel (*w*/*v*), first, 60 mg of LAP was dissolved in 12 mL of 1× sterile phosphate buffer solution (PBS, 0.01 M, and pH 7.2–7.4), and, after that, filtered. Then, 10 mL of the recently prepared 0.5% LAP solution was added into the vial containing 1 g of lyophilized GelMA and warmed in a water bath until the GelMA was properly dissolved (approximately 90 min at 40 °C). Finally, the whole solution was transferred into a sterile 5-mL syringe, which was used for the 3D printing of the scaffold.

### 2.2. Scaffold Design and 3D Printing Process

**Figure 1:**
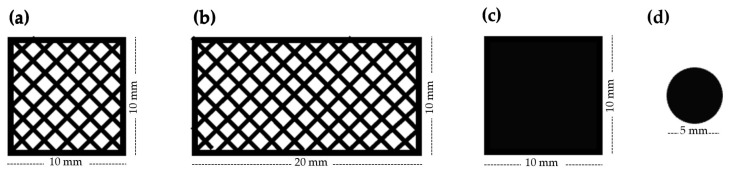
Design of the scaffolds used for (**a**) printability tests, (**b**) mechanical tests, (**c**) in vitro cell–biomaterial interaction analyses, and (**d**) in vivo assays.

The scaffolds were designed using the REGEMAT Designer software 1.5.1v2 and manufactured using a 3D bioprinter (REG4Life, REGEMAT 3D, Granada, Spain). Here, three 3D-printed designs were made (Figure 1). For the printability and mechanical characterization, meshed scaffolds with dimensions of 10 mm × 10 mm × 0.4 mm and 20 mm × 10 mm × 0.4 mm (w × l × h), respectively, with a pore size of 1.5 mm × 1.5 mm and a layer thickness of 0.2 mm, were designed (Figure 1a,b). For the in vitro cell–biomaterial interaction analyses, solid structures with 10 mm × 10 mm × 0.4 mm (w × l × h) dimensions and the same layer thickness were designed (Figure 1c). Finally, for the in vivo assay, 5-mm diameter and 400-µm thickness discs obtained from the in vitro scaffolds were used (Figure 1d), except for PLA, which was used the same scaffold as the in vitro assay, due to the impossibility of obtaining punches. The printing parameters of each biomaterial are listed in Table 1.

In addition to the printing process, the GelMA scaffolds were photopolymerized for hydrogel crosslinking and gelation. This procedure was conducted with a module of the REG4Life bioprinter with an excitation wavelength of 405 nm, from a distance of 5 mm, for 2 min, resulting in an irradiance of 66 mW/cm^2^.

### 2.3. Printability Tests

The extrusion capability and the absence of clogging has been analyzed during the printing process. In addition, the printing accuracy (shape fidelity and resolution) was evaluated by placing the 3D-printed scaffolds (Figure 1a) on a solid black surface with a millimetric rule. Macroscopic images of each of them were obtained using a Nikon SMZ 745T loupe equipped with a ProgRes CT3 digital camera (Nikon, Tokyo, Japan).

### 2.4. Mechanical Characterization

**Figure 2:**
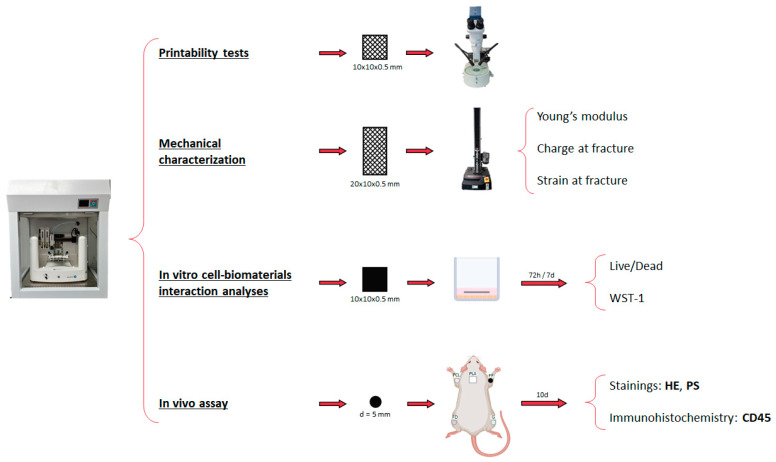
Scheme of the materials and methods employed in the printability tests, mechanical characterization, the in vitro cell–biomaterial interaction analyses, and the in vivo assay. HE refers to hematoxylin and eosin staining and PS to Picrosirius staining.

The mechanical evaluation was carried out with tensile tests, as previously described [36,37,38,39]. Briefly, an electromechanical material testing machine was used (Instron, Model 5943, Norwood, MA, USA), with a 50 N charge load (in case of PCL, FF, FD, and GelMA) and a 100 N charge load (in case of PLA). For this test, 6 scaffolds (Figure 1b) of each biomaterial were placed between the instrument clamps, leaving a constant distance of 10 mm, and tensile uniaxial stress was applied to the scaffolds until fracture was achieved, as can be seen in Figure 2. Young’s modulus was calculated as the tangent modulus slope of the initial linear portion of the stress–strain curve of each experimental trial, whereas the charge at fracture and the strain at fracture values were determined by selecting the point of the stress–strain curve when the fracture occurred.

### 2.5. In Vitro Cell-Biomaterial Interaction Analyses

The scaffolds’ cytotoxicity and cell metabolic activity were assessed with Live/Dead (L/D, Live/Dead^®^ Cell Viability Assay, Thermo-Fisher Scientific, Waltham, MA, USA) and water-soluble tetrazolium salt-1 (WST-1 assay, Roche, Grenzach-Wyhlen, Germany) assays, respectively. Both analyses were conducted according to manufacturers’ recommendations, as previously described [40,41], at 72 h and 7 days under standard cell culture conditions (37 °C and atmospheric air balanced with 5% CO_2_) (Figure 2). To determine the potential usefulness of these scaffolds in NTE, the human SK-N-AS cell line (ATCC, Manassas, VA, USA), a neural cell linage, was used. In this sense, 2 × 10^4^ cells in 300 μL of basal culture medium (Dulbecco’s modified eagle medium (DMEM) supplemented with 10% fetal bovine serum (FBS) and 1% antibiotic and antimycotic commercial solution (all products from Sigma Aldrich, Darmstadt, Germany)) were cultured in each well (24 well-plate). The next day, sterile 3D-printed scaffolds (Figure 1c) were placed on top of the cultured cells (day 0).

For the L/D assay, at each time, three scaffolds per biomaterial were used (n = 3). After 72 h and 7 days of cell culture, the scaffolds were removed, and the seeded cells were rinsed twice in PBS and then incubated with 300 μL of the working solution. After 15 min of incubation, the cells were rinsed in PBS and analyzed under a ZOE Fluorescent Cell Imager (BIO-RAD, Hercules, CA, USA).

Similarly, for the WST-1 assay, at each time, five scaffolds per biomaterial were analyzed (n = 5). After 72 h and 7 days of cell culture, the scaffolds were removed, and the seeded cells were incubated with 400 µL of WST-1 working solution for 4 h under standard culture conditions. Finally, three technical replicates were measured per sample.

In both cases, 2 × 10^4^ SK-N-AS, grown in basal medium, were used as 2D positive technical controls, whilst cells incubated in 2% triton X-100 (9036-19-5, Sigma-Aldrich, Darmstadt, Germany) in PBS were used as 2D negative technical controls. Additionally, wells with just culture medium were prepared as blanks of the WST-1.

### 2.6. In Vivo Assay

The laboratory animal procedures were conducted following the Spanish and European regulations for animal experimentation (EU directive No. 63/2020, RD 53/2013) and were approved by the local Animal Experimentation Ethical Committee No. 29/03/2022/052, Grant FIS P20/00318.

#### 2.6.1. Surgical Procedure

To determine the biocompatibility of the 3D-printed scaffolds, five adult male Lewis rats (JANVIER LABS, Laval, France) were used. The animals were housed in the Experimental Unit of the University Hospital Virgen de las Nieves (Granada), in individual plastic cages in a light- and temperature-controlled room (21 °C and 12 h light/dark), under veterinary and technical supervision, and with free access to food and tap water. Before surgery, the animals were anesthetized via intraperitoneal injection of a mixture of acepromazine (0.001 mg/g body weight (calmi-Neosan^®^, Labiana Life Sciences, Terrassa Spain)), ketamine (0.15 mg/g body weight (Imalgene 1000^®^, Boehringer Ingelheim, Tolouse, France)), and atropine (0.05 μg/g body weight (Pfizer, New York, NY, USA)). After that, the five types of 3D-printed scaffolds (Figure 1d) were implanted subcutaneously in each animal, as shown in Figure 2 (n = 5 for each biomaterial).

Finally, ten days after surgery, the animals were euthanized (by applying an anesthetic overdose), and the implanted scaffolds with the surrounding tissues (epidermis and dermal connective tissue) and healthy skin controls were harvested for histology.

#### 2.6.2. Histological Analyses

For the histological analyses, samples (experimental and controls) from each animal were fixed in 3.7% buffered formaldehyde solution for two days at room temperature, dehydrated, cleared, and paraffin-embedded [42,43]. Histological sections of 5 μm thickness were obtained, dewaxed, hydrated, and stained with hematoxylin and eosin (HE) for the evaluation of the general histological pattern. In addition, the Picrosirius histochemical method (PS) was applied to evaluate the collagen network around the implanted scaffold [44]. Furthermore, to assess the presence of immunological cells, the CD45 or leucocyte common antigen (ab10558, Abcam, 1:100 dilution in PBS) was evaluated by indirect immunohistochemistry following a previously described protocol [39]. All histological analyses were conducted with n = 5.

### 2.7. Quantitative and Statistical Analyses

To conduct the statistical analyses, the normality of the distribution of each variable was determined using the Shapiro–Wilk statistical test [45]. The tensile test, L/D quantification, and WST-1 results were non-normally distributed, therefore, the pairwise Mann–Whitney non-parametric test [46] was used to compare the Young’s modulus, charge at fracture, strain at fracture, cell viability index, and metabolic activity of each biomaterial (and with the positive and negative controls in the cases of cell viability index and metabolic activity).

The cell viability index was determined using the image processing software Fiji version: 2.14.0/1.54f (Fiji Is Just ImageJ, GitHub, San Francisco, CA, USA). First, the most representative L/D image of each sample was selected for the analyses (n = 3). Then, the area occupied by the green channel (live cells) and the red channel (dead cells) was determined. Finally, the cell viability index was calculated by dividing the area occupied by the green channel by the sum of the areas occupied by both channels. For the WST-1 results, the blank average value was subtracted from each condition, and the results were normalized relative to the positive control.

In this study, the results of each variable were expressed as mean ± standard deviation (SD) values, and data were analyzed using the Real Statistics Resource Pack software (Release 8.9.1) (Dr. Charles Zaiontz, Purdue University, West Lafayette, IN, USA, www.real-statistics.com, accessed on 7 November 2023). Moreover, *p* < 0.05 was considered statistically significant in all analyses.

## 3. Results

### 3.1. Printability

**Figure 3:**
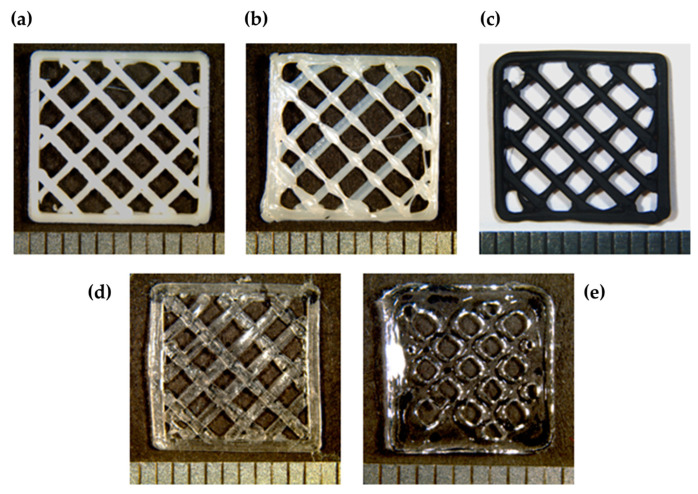
Macroscopic images of 3D-printed scaffolds of (**a**) PLA, (**b**) PCL, (**c**) FF, (**d**) FD, and (**e**) GelMA. Bright and contrast have been edited to improve their visualization.

All of the biomaterials showed great results in terms of printability (Figure 3). However, the PLA, FF, and FD scaffolds showed a higher shape fidelity and resolution than PCL, where it appears to have a higher quantity of biomaterial along the crossing zones, and GelMA, which expands before crosslinking, reducing the pore size. In addition, the printing time of the GelMA scaffolds was found to be considerably longer, although the thermoplastics required an increase in the bed temperature (Table 1) or the use of some additive products to improve their adherence to the bed. Additionally, the nozzle diameter when printing the FF scaffolds had to be changed from 0.4 to 0.8 mm, because the FF filament was getting stuck with the 0.4-mm nozzle, due to its high flexibility.

### 3.2. Mechanical Characterization

**Figure 4:**
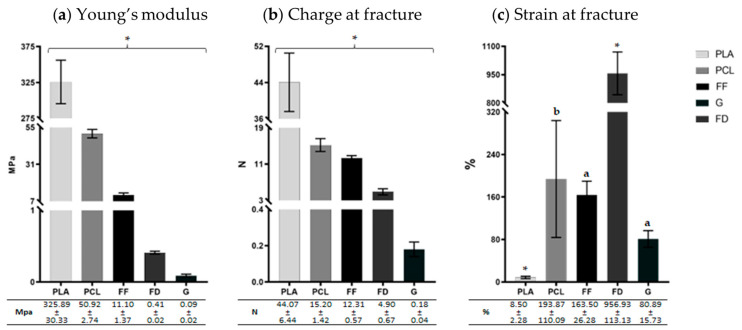
Graphic representation of tensile test results of PLA, PCL, FF, FD, and GelMA (G). (**a**) Young’s modulus (Mpa), (**b**) charge at fracture (N), and (**c**) strain at fracture (%). The results corresponding to each mechanical parameter are shown as mean ± standard deviation values. Statistically significant differences were determined with the Mann–Whitney test and represented as follows: ‘*’ indicates statistically significant differences (*p* < 0.05) between all biomaterials, ‘a’ indicates statistically significant differences (*p* < 0.05) between all biomaterials except “PCL,” and ‘b’ indicates statistically significant differences (*p* < 0.05) between all biomaterials except “FF” and “G.”

To determine the mechanical properties of PLA, PCL, FF, FD, and GelMA, the different scaffolds were subjected to tensile tests and the results have been summarized in Figure 4.

All groups obtained significantly different results (*p* < 0.05) from each other in both Young’s modulus and charge at fracture. In regard to the stiffness, PLA was the stiffer 3D-printed scaffold (325.89 ± 30.33 MPa), while FD and GelMA were softer (0.41 ± 0.02 MPa and 0.09 ± 0.02 MPa, respectively), as determined by their Young’s modulus (Figure 4a). Consequently, with respect to the charge at fracture (Figure 4b), the 3D-printed scaffolds showed similar behavior, with the PLA 3D-printed scaffolds presenting the highest resistance (44.07 ± 6.44 N), while GelMA exhibited the lowest resistance to fracture (0.18 ± 0.04 N). The differences between FD and PCL charge at fracture values are considerably lower than the differences between their Young’s modulus (FD had a Young’s modulus value 124.2 times lower than PCL but it resisted a charge at fracture, just 3.1 times lower). According to the strain at fracture (Figure 4c), FD obtained significantly higher results (956.93 ± 113.13%), followed by PCL, FF, and GelMA (the last two showed no significant differences with PCL). On the other hand, PLA revealed, significantly, the lowest capacity of deformation (8.5 ± 2.28%).

### 3.3. In Vitro Cell–Biomaterial Interaction Analyses

To evaluate the release of cytotoxic products from the 3D-printed scaffolds into the microenvironment, which could compromise the cell viability and functionality, the cell–biomaterial interaction was assessed with L/D and WST-1 assays.

**Figure 5:**
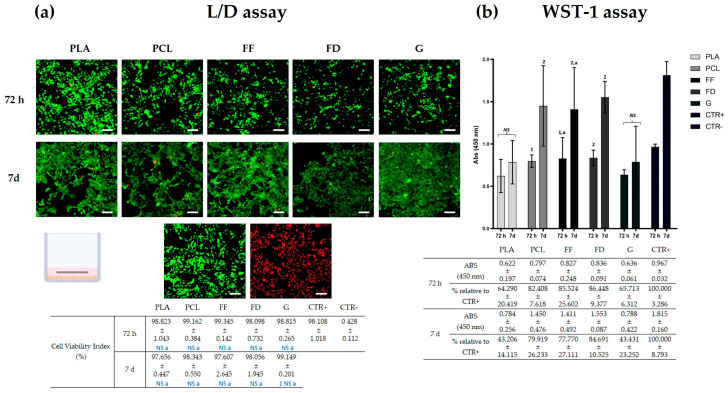
In vitro biocompatibility tests. (**a**) Representative panel of L/D assay of SK-N-AS seeded with PLA, PCL, FF, FD, and GelMA (G), and in 2D cultures as technical controls, after 72 h and 7 days of culture. Scale bar = 100 µm. (**b**) Graphic representation of WST-1 results of PLA, PCL, FF, FD, and GelMA (G). Statistically significant differences were determined with the Mann–Whitney test and are represented as follows: “NS” indicates no significant differences (*p* ≥ 0.05) between 72 h and 7 days; “2 or 1” indicates the number of biomaterials that obtained a significantly inferior cell viability (Figure 5a) or absorbance value (Figure 5b); and ‘a’ indicates no significant differences (*p* ≥ 0.05) with the CTR+ group.

The morphofunctional L/D test (Figure 5a), at 72 h, showed the presence of a similar number of viable cells within all of the printed scaffolds. According to the cell viability index, at 72 h of cell culture, there were no statistical differences (*p* > 0.05) between any of the groups, not even with the 2D positive control. Interestingly, after 7 days of cell culture, an increase in the number of viable cells within all biomaterials was observed, and results were more evident with the GelMA scaffolds. In addition, the cell viability index remained constant in all groups. However, GelMA exhibited a statistically significant mayor cell viability index compared to PLA (*p* < 0.05) after 7 days of cell culture.

In regard to the WST-1 assay (Figure 5b), the cells seeded with PCL, FF, and FD scaffolds showed a significantly greater metabolic activity than those seeded with PLA and GelMA (*p* < 0.05), both after 72 h and after 7 days of cell culture. Furthermore, these three groups were the only groups that significantly increased these metabolic activities (*p* < 0.05) after 7 days of cell culture, whereas the cells seeded with PLA and GelMA did not.

### 3.4. Histology of Implanted 3D-printed Scaffolds

To assess the biocompatibility of the 3D-printed scaffolds and their interaction with a host species, they were subcutaneously implanted (back and limbs) in adult rats, and the host response and presence of immunogenic cells in the surrounding tissue were evaluated histologically after 10 days of implantation.

**Figure 6:**
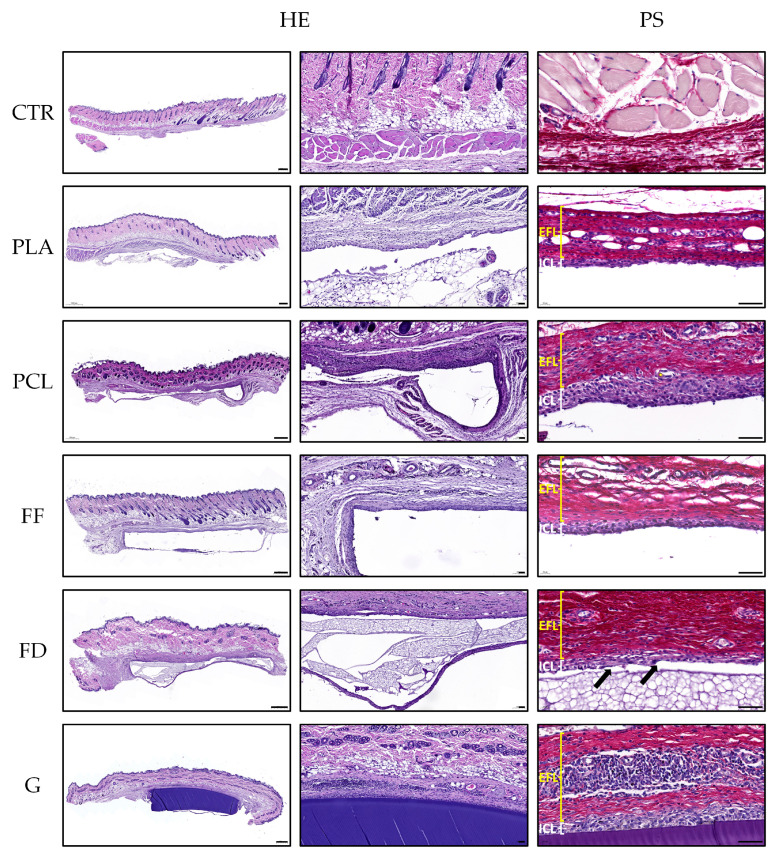
Representative panel of the hematoxylin and eosin (HE) and Picrosirius (PS) staining of the in vivo samples of PLA, PCL, FF, FD, GelMA (G), and healthy rat skin tissue (CTR). The PS images distinguish an external fibrotic layer (EFL) and an inner cellular layer (ICL). The black arrows indicate syncytial formations. Scale bar of the first column indicates 500 µm, while the scale bar of the second and third columns indicates 50 µm.

Due to technical limitations, only FD and GelMA scaffolds could be embedded in paraffin, while the other biomaterials had to be removed after tissue fixation and prior tissue processing. Consequently, the histological evaluation primarily focused on the surrounding connective tissue or pseudocapsule formed by the host animals. (Figure 6). Nevertheless, FD and GelMA, which were maintained after histological processing, allowed the evaluation of the histological features of the implanted scaffolds. In the case of FD, a microscopic analysis revealed a good preservation of the biomaterial with a porous structure. The GelMA 3D-printed scaffolds, on the other hand, were fully conserved during the period evaluated, with a highly consistent and homogeneous structure.

When the host response was evaluated, in general, the presence of a well-defined pseudocapsule composed of an inner cellular layer (ICL) and an external fibrotic layer (EFL) was observed around all of the grafted scaffolds, with slight differences. The pseudocapsule formed around the PLA and FF 3D-printed scaffolds was thinner than that observed around the FD, PCL, and GelMA scaffolds; however, in all cases, an important amount of collagen fibers was observed with PS staining. Interestingly, the ICL was thicker around the GelMA scaffolds, followed by the PCL and FD scaffolds, while in the FF and PLS scaffolds it was considerably thinner. Moreover, the histology confirmed that the cells observed within the formed pseudocapsules corresponded, in all cases, to a mononuclear infiltration and, therefore, lymphocytes and macrophages, with some syncytial formations or giant cells observed around the FD scaffolds (Figure 6, black arrows). In addition, a large amount of blood vessels was observed within the pseudocapsule wall, and some blood vessels contained a variable amount of perivascular mononuclear infiltration, as can be seen, for instance, in the external fibrotic layer of the GelMA histological images.

**Figure 7:**
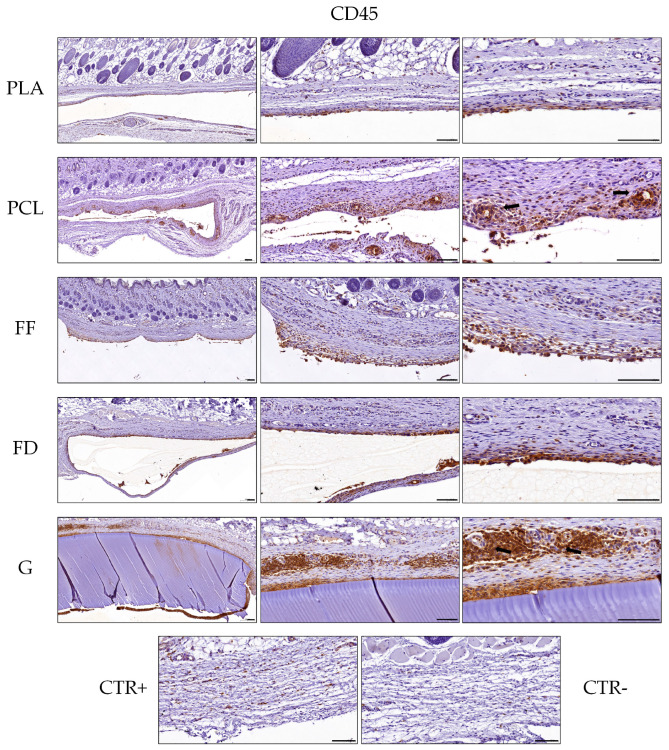
Representative panel of CD45 immunohistochemistry of the in vivo samples of PLA, PCL, FF, FD, GelMA (G), and healthy rat skin tissue (incubated with CD45 antibody (CTR+) and without CD45 antibody (CTR−). The black arrows indicate signs of perivascular infiltration. Scale bar = 100 µm.

In this study, the CD45 protein, or common leucocyte antigen, was evaluated by indirect immunohistochemistry to determine the presence of white blood cells (Figure 7). These immunohistochemical analyses revealed that most of the cells observed within the ICL of the pseudocapsules were of the immunological lineage. Thus, this immunostaining confirmed the histological findings described above and the fact that more mononuclear infiltration was observed around the GelMA and PCL 3D-printed scaffolds than the other groups, with these cells being less abundant around PLA. Moreover, this analysis also confirmed the perivascular infiltration (Figure 7, black arrows) and syncytial cells (Figure 7). All of these findings corroborated a local host inflammatory response with some features of a foreign body reaction around the grafted 3D-printed scaffolds. Finally, no signs of polymorphonuclear infiltration or the presence of a plasma-cell-mediated reaction were observed around the biomaterials studied during the period analyzed.

## 4. Discussion

In this in vitro and in vivo study, we have described the generation and characterization of different 3D-printed scaffolds composed of PLA, PCL, FD, FF, and GelMA biomaterials for NTE applications. These 3D-printed scaffolds were structurally and mechanically characterized, and the cell–biomaterial interactions were determined by using the neural cell line SK-N-AS in vitro. Additionally, the in vivo biocompatibility of the 3D-printed scaffolds was histologically determined after 10 days of subcutaneous implantation in rodents.

The ideal scaffold for NTE should mimic the structural and mechanical properties of neural tissue because it has been shown that structural and mechanical mismatch between the engineered tissue and the host tissue may cause additional implant-induced damage [47]. Therefore, in recent years, the application of 3D printing technology has been used for the development of diverse neural scaffolds that mimic some of the main features of neural tissue [48,49,50]. Indeed, 3D printing technology enables the generation of organ-like constructs with high resolution and reproducibility; however, to achieve that, the printability of the used biomaterials needs to be meticulously examined [51]. An optimal printability is characterized by adequate extrudability, shape fidelity, and the proper standardization of the printing process, which are the main complications associated with these properties’ nozzle clogging, non-continuous deposition, and flawed retraction [52]. On the one hand, when dealing with thermoplastics, it is possible to address these first two complications by just adjusting the nozzle diameter, flow speed, and printing temperature. However, in the case of PCL, the deposited material quantity was found to be less uniform, which affects the shape fidelity of the 3D-printed scaffolds. On the other hand, when dealing with hydrogels, like GelMA, it is essential to achieve an optimal viscosity by adjusting the hydrogel composition and printing temperature, since a low viscosity could lead to the deformation and collapse of the scaffold during printing, and a high viscosity could result in nozzle clogging [53]. Additionally, GelMA requires photoinitiated radical polymerization, after 3D printing, to form covalently crosslinked hydrogels and, therefore, to gain consistency [54]. Since GelMA hydrogels are not crosslinked during the 3D printing process, but afterward, they slightly expand on the printing bed, reducing the pore size, and thus compromising the resolution of the scaffold. The ability to perform an optimal retraction depends mainly on the 3D printer hardware rather than on the used biomaterials, and it is essential for controlling over-extrusion during high-precision printing [55]. Mechanical-extrusion-based 3D printing was used because it offers advantages in terms of controlling the retraction compared to pneumatic-extrusion-based 3D printing, which is connected to a pressure source that creates certain delays between the commands given and what the hardware does [56]. However, it was not possible to completely avoid a flawed retraction, which has led to the accumulation of biomaterial at various points on the 3D-printed scaffolds, affecting their shape fidelity, especially in the case of PCL and GelMA.

To determine the mechanical properties of the 3D-printed scaffolds, they were subjected to tensile tests. Thus, it was possible to evaluate their ability to withstand stretch deriving from the formation of external fibrotic tissue, and if they are easy- to manipulate for clinicians [15,57]. Previous studies [37,58,59] have indicated that the Young’s modulus of rat sciatic nerves varies between 10.19 and 18.66 MPa, which coincides with the Young’s modulus values of FF scaffolds. The PLA and PCL scaffolds showed significant stiffness, which could potentially affect the nerve tissue structure and neuronal networks; however, this it could be addressed by blending these polymers with softer biomaterials to achieve more suitable viscoelastic properties [60]. The FD and GelMA scaffolds, on the other hand, have exhibited higher viscoelastic properties than the rat native nerves previously measured, while keeping proper shape fidelity and manageability. This makes them promising biomaterials for the generation of spinal cord substitutes, since the spinal cord Young’s modulus varies between 0.012 and 1.37 MPa [61]. In relation to strain at fracture, all thermoplastics, except for PLA, obtained higher values of strain at fracture than the rat native nerves (56.97 ± 4.68%) previously measured [58] and human spinal cord maximum strain (~10%) [62], which shows their elastic properties. Furthermore, FD showed an unproportioned resistance to fracture in relation to its viscoelastic properties. This suggests that, despite its excellent viscoelastic properties, FD can withstand relatively high forces, which is a valuable biomechanical property for NTE. However, it is important to consider that it has been tested as rectangular, and not tubular, scaffolds. Therefore, when generating tubular scaffolds with these biomaterials, some minimal variations in the biomechanical properties may occur, and a new mechanical evaluation should be carried out. In this sense, it would be of interest to subject the tubular 3D-printed scaffolds or novel organ-like 3D-printed structures to compression or rheological tests, since neural tissues are naturally subjected to compression and torsion forces [63].

The human SK-N-AS neural cell line was used to determine the biocompatibility of the different scaffolds, since they are related to the nerve tissue and share surface proteins and other biomarkers with neural cells [64]. Moreover, they have a high in vitro proliferation capacity. The cell proliferation, viability, and activity were evaluated using L/D and WST-1 assays, after 72 h and 7 days of cell culture, to understand how these biomaterials could affect cellular behavior not instantly but gradually. The in vitro biocompatibility of PLA and PCL has been tested previously by other research groups [22,65,66], but not with the SK-N-AS cell line. In this case, both of these thermoplastics obtained great results of the cell viability index in the in vitro L/D assay at 72 h and 7 days of cell culture, as expected from previous studies [67,68]. The non-toxicity and cell viability index of FF with a neural cell line have been examined here for the first time, obtaining similarly good results as the other biomaterials. FD, on the other hand, has already been used for cell culture in microfluidics [34,69], but its cell viability index had never been evaluated in a neural cell line until now. It has achieved, as FF, comparable results with the other biomaterials. The GelMA hydrogel comes from gelatin, an irreversible partial degradation product of collagens, which has been modified with methacrylic acid so that it can be crosslinked and, thus, maintain the hydrogel consistency at 37 °C and improve its mechanical and structural properties [70]. Therefore, as they come from collagen, a natural and highly conserved protein, they are less likely to liberate cytotoxic particles and to affect the cell viability. As expected, GelMA has been shown to be the biomaterial with a greater cell viability index after 7 days of cell culture, according to the quantification of the L/D assay. However, the differences with the other biomaterials, except for PLA, are not statistically significant, which is probably due to the early endpoint, which may have caused the differences in long-term cell viability caused by the liberation of cytotoxic particles to not become visible. With regard to the WST-1 results, it is important to note that, here, we are inferring cell viability using bulk metabolic measurements from all of the cells in a population, and not directly counting the individual live and dead cells in a population, like in the L/D assay, which could lead to technical misinterpretations [71]. For instance, it is surprising that GelMA, which has demonstrated great results in terms of the cell viability index in the L/D assay, obtained these low values of metabolic activity. One explanation could be that, due to the saturation of the sample after 7 days of cell culture, the metabolic activity of the population may have been reduced. This occurs similarly with PLA, although the cell population does not seem to be as massive as that observed in the GelMA group. It is not easy to distinguish between proliferative arrest and cell death when using a bulk metabolic assay, so, in this case, considering the L/D results, it is possible that the PLA is causing a proliferative arrest, reducing the metabolic activity without affecting the cell viability.

An implanted scaffold is considered biocompatible if it does not cause an intolerable inflammatory response that is disproportionate to its beneficial effects [72]. Therefore, an in vitro study is not sufficient to determine the biocompatibility of the different scaffolds. Thus, an in vivo study was carried out to analyze the possible undesirable host rejection through the assessment of the general morphology of the surrounding tissue, the fibrotic stromal reaction, and the presence of inflammatory cells associated with the various scaffolds. The scaffold implantation procedure induces an inflammatory response to prevent tissue damage, insulate and eliminate the foreign material, and start the tissue repair process [73]. The first hours or days are characterized by microvasculature and tissue damage, which leads to the migration of neutrophils and monocytes towards the implant. This process enhances the inflammation and induces angiogenesis, which is essential for the generation of a granulation tissue and the successful integration of the implant. Then, these cells differentiate into macrophages, which form a continuous layer around the biomaterial, leading to the development of a highly fibrous and avascular capsule [74].

PLA is a type of lactic acid derivative produced from renewable resources that has been approved by the U.S. Food and Drug Administration (FDA) and European regulatory authorities for biomedical use [75]. After 10 days of its implantation, the formation of an external fibrotic layer composed primarily of collagens can be observed, similar to that seen after 12 weeks of implantation in another study [76], which makes us consider that the generation of this fibrotic tissue actually occurs earlier than expected. On the other hand, the inner cellular layer surrounding the pseudocapsule is less dense than that previously reported [76], which is probably due to the later endpoint of the in vivo study (12 weeks instead of 10 days), but also less dense than the other biomaterials of this study, which indicates a lower acute inflammatory response. PCL has been safely used in biomedical science for more than 70 years, from sutures to tissue and organ replacement by 3D printing [77]. This biomaterial has also induced an in situ synthesis of collagen fibers, which was expected from other studies [78,79]. In addition, PCL showed the greatest acute inflammatory response, with a dense layer of inflammatory cells surrounding the pseudocapsule, which was even more severe than FF and FD, biomaterials whose in vivo biocompatibility had not been tested before. This was probably caused by the leaching of low molecular mass compounds, either through degradation or because of the presence of leachable impurities [80]. The in vivo biocompatibility of FF and FD had not been tested before, and they have obtained comparable results to PLA in terms of the alteration of the matrix and immunological response in this study. Additionally, the periphery of the FD scaffolds is characterized by the presence of multiple small blood vessels, which may be attributed to the activity of M1 macrophages (classically activated phenotype) during their crucial role in the early stages of vascularization [81]. GelMA, as mentioned before, is a natural hydrogel derived from collagen that retains a variety of natural cell-binding motifs such as arginine–glycine–aspartic acid (RGD) and matrix metalloproteases, which are required to support cell adhesion, proliferation, and migration [82]. In contrast, the GelMA scaffolds induced the formation of a dense immunological cell layer at the periphery of the pseudocapsule and no infiltration of cells into the hydrogel. One possible explanation is that the high crosslinking intensity used to improve the mechanical properties of the GelMA scaffolds may have altered their porosity and the presence of bioactive motifs [83], affecting, consequently, the cell–biomaterial interaction and their biocompatibility.

Biodegradability, in the context of NTE, is an essential characteristic for the development of optimal scaffolds. First, biodegradable scaffolds eliminate the need for surgical removal, as they are absorbed by the surrounding tissue. Additionally, they support the reparation of the injury as they are being biodegraded, helping the neighboring cells to produce their own extracellular matrix [84]. Moreover, the degradation rate of the scaffold should match the regeneration rate of the tissue, as this would allow for the most beneficial healing [85]. The biodegradability of the PLA, PCL, and FF scaffolds could not be assessed, due to technical limitations that prevented their preservation during sample processing. However, when the FD and GelMA scaffolds were evaluated after 10 days of implantation, no signs of cell infiltration or loss of material were observed. Thermoplastics have, in general, slow degradation rates [86]. For instance, the biodegradation time to complete the mass loss for PLA has been reported as greater than 12 to 16 months [87], and PCL has not shown signs of visible biodegradation until 6 months, when applied in vivo [88]. Therefore, in the case of FD, the biodegradability tests should be assessed after longer periods of implantation. The biodegradation rate of GelMA scaffolds depends on multiple factors, including the GelMA concentration, the type and concentration of the photoinitiator, the crosslinking conditions, and the geometry of the scaffold [89]. However, previous studies have not reported signs of biodegradation until after one month of in vivo implantation [53].

Although promising in vivo biocompatibility results have been reported, with no severe immune response and no evidence of necrotic tissue, it is important to consider biocompatibility not only from a short-term perspective, but also in the longer term [80]. The study of the long-term biodegradation of materials at the implant site into safe components that will eventually be eliminated from the body will be of vital importance [72]. Furthermore, in vivo nerve and spinal cord repair studies are still required to validate the capacity of the reparation and regeneration of these novel biomaterials in the NTE field.

## 5. Conclusions

All of the biomaterials tested in this study showed promising results for NTE applications, with desirable mechanical properties for peripheral nerves or the spinal cord, great in vitro cell–biomaterial interaction, and adequate in vivo biocompatibility. Moreover, in vivo nerve and spinal cord repair studies are still needed to validate the therapeutic capacity of the engineered substitutes made of these biomaterials. The thermoplastics PLA and PCL showed great results in the in vitro cell–biomaterial interaction analyses and in the in vivo biocompatibility assay, as expected, although it would be interesting to study their combination with other thermoplastics (FF and FD, for example) or hydrogels like GelMA to reduce their rigidity and enhance their viscoelastic properties before testing neural engineering or used to generate nerve guide conduits constituted by an external meshed scaffold and an intraluminal pro-regenerative filler. The novel thermoplastics FF and FD demonstrated great mechanical properties for NTE. Additionally, the in vivo biocompatibility of these biomaterials has been assessed here, for the first time, and they have obtained promising results. However, their long-term biodegradation should be tested in order to analyze the liberation of toxic products. Finally, it is crucial to study how the crosslinking intensity and functionalization strategies could improve GelMA’s structural and biological properties for potential application as an intraluminal filler of nerve guides, where its stiffness and tensile strength would not be as necessary.

## Figures and Tables

**Table 1:** Printing parameters optimized for the 3D printing of each biomaterial. “Flow speed” refers to the extrusion speed, while “infill speed” refers to the speed of the printhead along the design. The inner nozzle diameter and type of extruder have been also specified in corresponding columns.

Material	Printing Temperature (°C)	Flow Speed (mm/s)	Infill Speed (mm/s)	Nozzle Diameter (mm)	Extruder
PLA	220	1.00	8	0.40	Filament
PCL	80	1.00	8	0.40	Filament
FF	249	1.00	6	0.80	Filament
FD	230	1.75	3	0.40	Pellet
GelMA	21–22	1.50	6	0.41	Syringe 5 cc

## Data Availability

The data supporting the reported results can be found using the DOI 10.5281/zenodo.10903694.
